# Advanced muscle imaging in adolescent elite rowers utilizing diffusion tensor imaging: Association of imaging findings with stroke typology

**DOI:** 10.1371/journal.pone.0294693

**Published:** 2023-11-29

**Authors:** Adrian Alexander Marth, Timo Alexander Auer, Gergely Bertalan, Pimrapat Gebert, Timo Kirchenberger, Dominik Geisel, Bernd Hamm, Sarah Keller

**Affiliations:** 1 Department of Radiology, Charité—Universitätsmedizin Berlin, Corporate Member of Freie Universität Berlin and Humboldt-Universität zu Berlin, Berlin, Germany; 2 Clinician Scientist Program, Berlin Institute of Health at Charité—Universitätsmedizin Berlin, Berlin, Germany; 3 Institute for Biometry, Charité—Universitätsmedizin Berlin, Corporate Member of Freie Universität Berlin and Humboldt-Universität zu Berlin, Berlin, Germany; 4 Charité—Universitätsmedizin Berlin, Corporate Member of Freie Universität Berlin and Humboldt-Universität zu Berlin, Berlin, Germany; Loughborough University, UNITED KINGDOM

## Abstract

**Purpose:**

Muscular overuse injuries are a common health issue in elite athletes. Changes in the muscular microenvironment can be depicted by Diffusion Tensor Imaging (DTI). We hypothesize that the biomechanics of different stroke typologies plays a role in muscle injury and tested our hypothesis by magnetic resonance imaging (MRI) examination of the lumbar spine muscles of adolescent rowers utilizing DTI.

**Methods and materials:**

Twenty-two male elite rowers (12 sweep, 10 scull rowers) with a mean age of 15.8 ± 1.2 years underwent 3-Tesla MRI of the lumbar spine 6 hours after cessation of training. Apparent diffusion coefficient (ADC) and fractional anisotropy (FA) were calculated for the erector spinae and multifidus muscle. Student’s t-test was used to test differences of DTI parameters between sweep and scull rowers and a Pearson correlation was utilized to correlate the parameters to training volume.

**Results:**

ADC values in the erector spinae and multifidus muscle were significantly higher (p = 0.039) and FA values significantly lower (p < 0.001) in sweep rowers compared to scull rowers. There was no significant association between DTI parameters and training volume (r ≤ -0.459, p ≥ 0.074).

**Conclusions:**

Our DTI results show that lumbar spine muscle diffusivity is higher in sweep rowers than in scull rowers. Altered muscle diffusivity is suggestive of microscopic tissue disruption and might be attributable to biomechanical differences between stroke typologies.

## Introduction

Muscular overuse is a common phenomenon in elite rowers and can result in muscular fatigue, pain, and decreased performance [1; 2]. The etiology of muscular overuse in rowers is multifactorial and can involve factors such as training load, technique, equipment, biomechanical factors and individual characteristics of the athlete [3; 4]. Especially in fatigued muscles, the repetitive movements performed while rowing can alter spinal loading patterns and joint mechanics [5; 6].

In rowing sports, the movement consists of four distinct phases: the catch, drive, finish, and recovery [1]. Two different stroke types can be distinguished. Scull rowers move the boat with two oars, one on each side, while sweep rowers only use one oar, which can be placed on the bow side or stroke side of the boat. A study of Strahan et al. investigating differences in spinopelvic rowing kinematics showed that sweep rowers have a greater lateral bend of the trunk throughout the whole rowing movement and greater axial rotation at the catch compared to scull rowers [7].

A clinical diagnosis of muscle injury is often confirmed by a magnetic resonance imaging (MRI) examination, which will detect fiber tears or intramuscular hemorrhage [8]. However, accurate diagnosis remains challenging, and conventional MRI might be inconclusive in cases of minor tears or fatigue-induced injury [9].

Various quantitative MRI techniques are available to visualize the muscular microstructure [10]. Among these, Diffusion Tensor Imaging (DTI) is an MRI technique that is based on findings initially reported by Cleveland et al. in the 1970s [11] and relies on the correspondence between cell geometry and the anisotropic nature of water diffusion in muscular tissue [12]. Therefore, DTI is sensitive to early pathophysiological tissue changes by not only quantifying water diffusion but also reflecting its directional properties through description of a tensor decomposition [13]. This tensor can be diagonalized by analysis of eigenvalues (*λ1*, *λ2*, *λ3*), which reflect principal effective diffusivity [14]. Eigenvalues can be summarized (trace, *λ1+λ2+λ3)* or reported as mean diffusivity known as apparent diffusion coefficient (ADC, trace/3). Diffusion asymmetry can be described by the fractional anisotropy (FA), which varies between 0 and 1, forming a sphere for the value 0 in perfect isotropic diffusion and progressing to 1 with advancing eigenvalue inequivalence.

DTI is influenced by factors such as acquisition parameters and demographic data [15; 16], but has already been used in clinical settings such as in the assessment of idiopathic myopathies [17]. Experimental studies investigating DTI and muscle pathophysiology have confirmed that muscle injury leads to higher tissue diffusivity and disorganized fiber structure [18]. Moreover, these changes could be directly correlated to the amount of histological damage such as interstitial fluid accumulation and proportion of damaged cells [19]. In sports medicine, DTI has been utilized to monitor the microstructural muscular changes after strenuous exercise (e.g. long-distance running) [20] and to assess muscle injury recovery as well as the effect of preventive exercises [21; 22]. Other authors suggest that an increased diffusivity is related to eccentric muscle contractions, which can also damage the microstructural muscular structure [23–25].

Therefore, we hypothesized that DTI may assess subclinical muscle injury and investigated for variations in DTI metrics between sweep and scull rowers.

## Material and methods

### Study design and participants

This cross-sectional single-center study was approved in 12/2020 by the local ethics committee (Ethics Committee of Charité University Medicine Berlin, approval number EA 2/180/20). After approval, participants were recruited through a cooperation with a local rowing club from 01/2021 until 02/2021. Written informed consent was obtained from all participants and participants’ parents and legal guardians. All study procedures were performed in accordance with the ethical principles of the Declaration of Helsinki. During data collection, the authors had access to information that could identify individual participants.

Examinations were performed 6 hours after the end of training on water (2 hours) and ergometer training (0.5 hours) on four consecutive weekends in February and March 2021. All participants underwent the same training regimen at the same intensity level. For this study, all rowers who competed at state or national level were included. Exclusion criteria comprised a known history of chronic musculoskeletal disease (e.g. low back pain), known history of interventional or surgical procedures of the spine, and general contraindications to MRI such as history of pacemaker implantation.

Participants filled out a questionnaire to obtain basic anthropometric data and information on training volume, type of training, and stroke typology.

### Magnetic resonance imaging

Participants underwent imaging on a 3-Tesla MR scanner (MAGNETOM Skyra; Siemens Healthineers, Erlangen, Germany) using the built-in whole-body coil of the patient table. Participants were imaged in supine position (head first) using a clinical protocol for lumbar spine examination.

The clinical protocol included a transverse fat-suppressed T2-weighted turbo spin-echo (TSE) sequence with inversion recovery, a T1-weighted 3D volumetric interpolated breath-hold examination sequence with Dixon fat suppression and readout-segmented echo-planar diffusion weighted imaging for DTI calculation. Imaging parameters were as follows: Fat- suppressed T2-weighted imaging with inversion recovery: transverse field of view: 400 × 324 mm; 50 slices; image size: 320 × 240 voxels with a voxel size of 1.25 × 1.35 × 3 mm; slice gap: 0.6 mm; TE 44 ms; TR: 7000 ms; inversion recovery time: 220 ms; flip angle: 150°; readout acceleration factor of two; bandwidth: 252 Hz/pixel. T1-weighted Dixon: transverse field of view: 400 × 324 mm; 96 slices; image size: 320 × 240 voxels with a voxel size of 1.25 × 1.25 × 3 mm; slice gap: 0.6 mm; TE: 1.26 ms; TR: 4.06 ms; flip angle: 9°; Cartesian k-space trajectory, and bandwidth of 1040 Hz/pixel. DTI was performed with a fat-suppressed 2D RESOLVE sequence: transverse field of view: 200 × 200 mm, 18 slices; image size: 80 × 80 voxels with voxel size of 2.5 × 2.5 × 5 mm; slice gap: 1.0 mm; TE: 48 ms; TR: 3000 ms; flip angle: 180°; three readout segments; readout acceleration factor of two and b-values of 0 and 400 s/mm^2^ measured in 12 diffusion directions. The total acquisition time of the protocol was 15 minutes and 35 seconds.

### Data analysis

DTI parameters (ADC and FA) were calculated using syngo.via client imaging software (Neuro 3D plugin, software release V30A, Siemens Healthineers, Erlangen, Germany).

All quantitative and qualitative image analyses were performed in consensus by two radiologists (AAM and SK) with 4 and 10 years of experience in musculoskeletal imaging who were blinded to participant data and analyzed images in random order on a dedicated picture archiving and communication system under certified reading room conditions.

**Fig 1:**
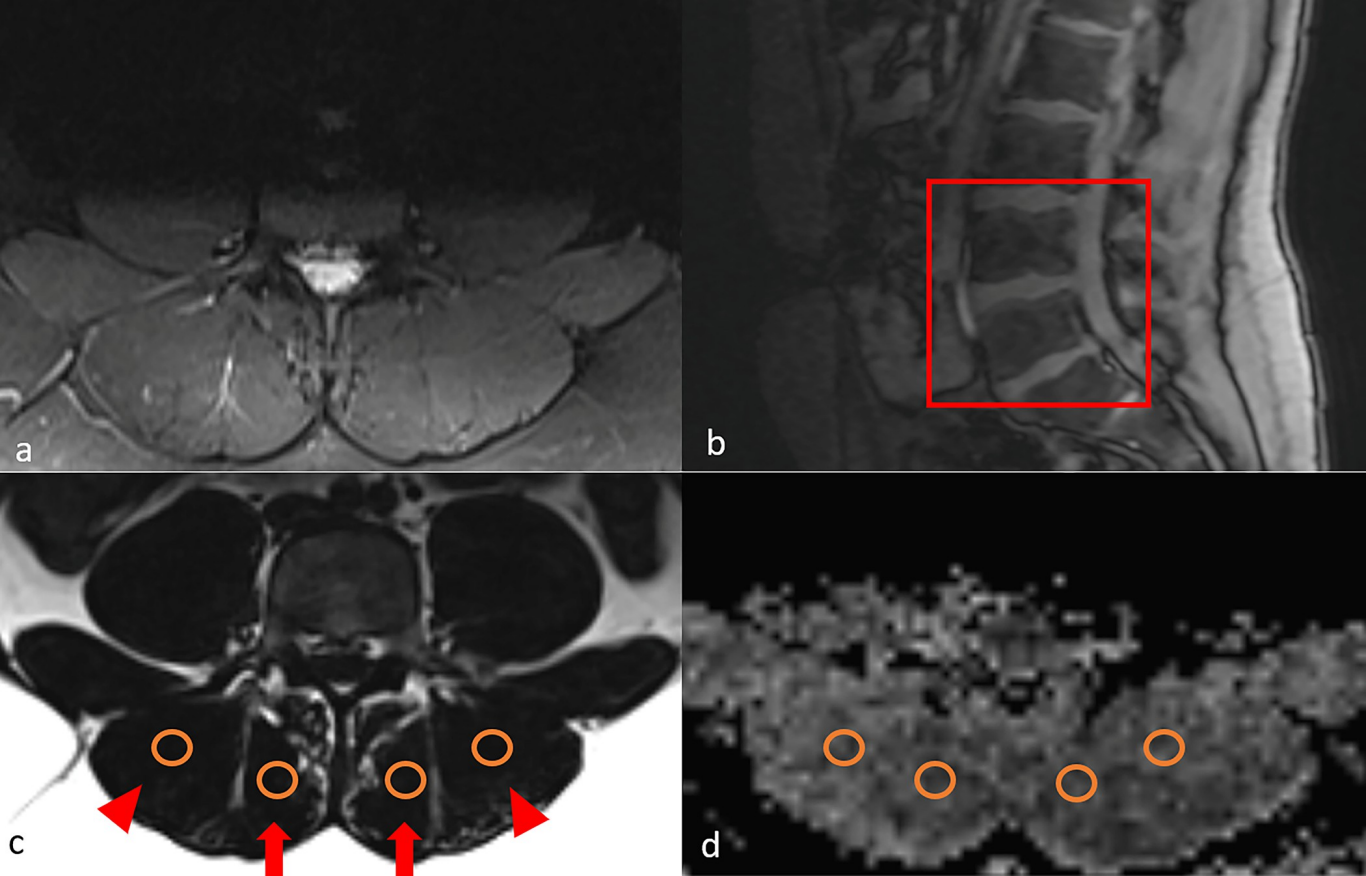
Magnetic resonance imaging analysis. Qualitative image analyses were performed on fat-suppressed T2-weighted images (a) and T1 Dixon images (c). Regions of interest (ROIs) were seeded from the upper vertebral endplate level of the L4 vertebra to the lower endplate level of the L5 vertebra (b). Exemplary ROI seeding on fat-only T1 Dixon images in the erector spinae muscle (arrowheads) and multifidus muscle (arrows) (c). ROIs were then transferred to the corresponding tensor maps (d).

Regions of interest (ROIs) with a size of 10 mm^2^ were seeded bilaterally in the fat-only Dixon images of the erector spinae muscle and multifidus muscle, respectively. These were placed at central L4 level, at the disc level of L4/5 and at central L5 level, avoiding vascular areas and regions with fatty infiltration (**Fig 1**). Subsequently, all 12 ROIs for each subject were transferred to the corresponding ADC and FA maps for further analysis. Qualitative image analysis was conducted on inversion-recovery and Dixon-based images.

### Statistical analysis

Statistical analysis was performed using SPSS Statistics (Software release v27, IBM, Armonk, USA). Patient characteristics are expressed as mean ± standard deviation. Normal distribution was confirmed using the Shapiro-Wilk test. Homogeneity of variances was tested and confirmed with the Levene-Test. Student’s t-test was used to indicate differences between variables of sweep and scull rowers. For correlations between DTI parameters to training characteristics, a Person correlation with Bonferroni correction for multiple testing was conducted. Statistical testing was done within an exploratory framework at a two-sided significance level of α = 0.05.

## Results

### Anthropometric data and training characteristics

A total of 22 male-only participants with a mean age of 15.8 ±1.2 years were enrolled (12 sweep rowers and 10 scull rowers). Their average training volume was 16.0 ±3.3 hours per week. Anthropometric data did not differ between both stroke typology groups (**Table 1**). MRI examinations were performed at a mean time of 6.2 ± 0.3 hours after cessation of training.

**Table 1:** Participant demographics and training specifics.

	Total study group (n = 22)	Sweep rowers (n = 12)	Scull rowers (n = 10)	*p*-value^*2*^
Age (years)^1^	15.8 ±1.2	15.3 ± 0.9	16.4 ± 1.5	0.535
Height (cm)^1^	185 ± 8	187 ± 6	182 ± 9	0.698
Weight (kg)^1^	80.8 ± 9.4	82.1 ± 8.6	77.5 ± 7.9	0.356
BMI (kg/m^2^)^1^	23.6 ± 2.2	22.8 ± 1.9	24.1 ± 2.3	0.886
Training volume / week (hours)^1^	16.0 ± 3.3	15.5 ± 2.5	17.0 ± 3.0	0.815

^1^Data are given as mean ± standard deviation

^2^p-value by Student’s t-test for comparison of sweep and scull rowers

BMI, Body Mass Index

### Magnetic resonance imaging

The qualitative analysis of images found no evidence of fatty muscle infiltration, fractures, spinal ligament abnormalities, or abnormal signal alterations suggestive of muscle edema. **Table 2** summarizes the results for the DTI parameters of the entire study population, as well as the sweep and scull rowers.

**Table 2:** Mean diffusion tensor imaging parameters of the erector spinae and multifidus muscles in the total study population and separately for sweep rowers and scull rowers.

Parameter	Total study group (n = 22)	Sweep rowers (n = 12)	Scull rowers (n = 10)	*p*-value^2^
ADC^1^ (mm^2^/s)	1.897 ±0.106	1.969 ± 0.096	1.854 ± 0.089	0.039*
FA^1^	0.238 ± 0.029	0.207 ±0.038	0.261 ± 0.027	<0.001*

^1^Data are given as mean ± standard deviation

^2^p-value by Student’s t-test for comparison of sweep and scull rowers

* denotes statistical significance

ADC, apparent diffusion coefficient; FA, fractional anisotropy

**Fig 2:**
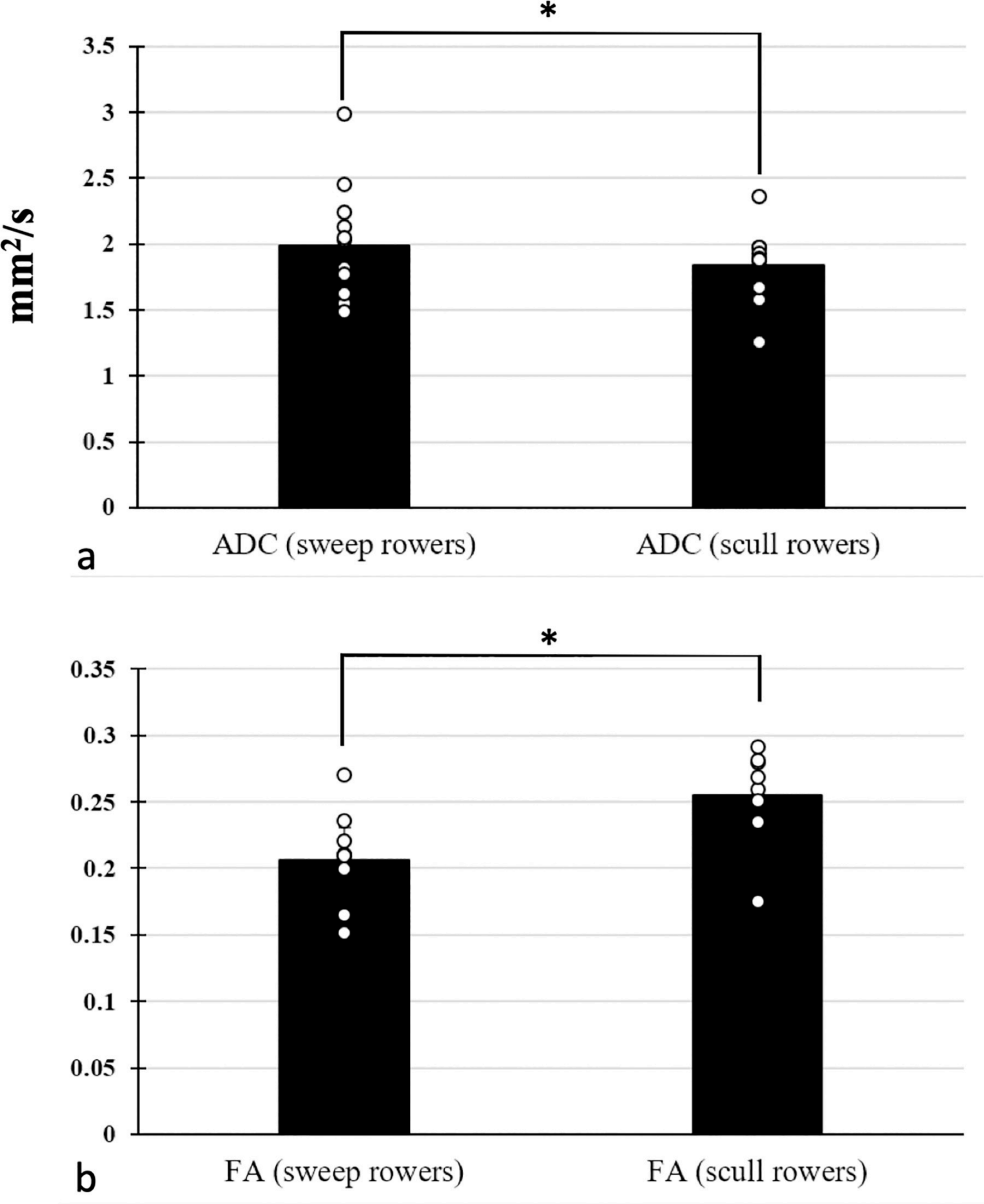
ADC and FA values for sweep and scull rowers. ADC values (a) were significantly higher and FA values (b) significantly lower in sweep rowers. ADC, Apparent Diffusion Coefficient; FA, Fractional Anisotropy.

ADC values were significantly higher and FA values significantly lower in sweep rowers compared to scull rowers (ADC: 1.969 ± 0.096 mm^2^/s vs. 1.854 ±0.089 mm^2^/s, p = 0.039 and FA: 0.207 ±0.008 vs. 0.261 ±0.017, p <0.001, **Fig 2**).

**Fig 3:**
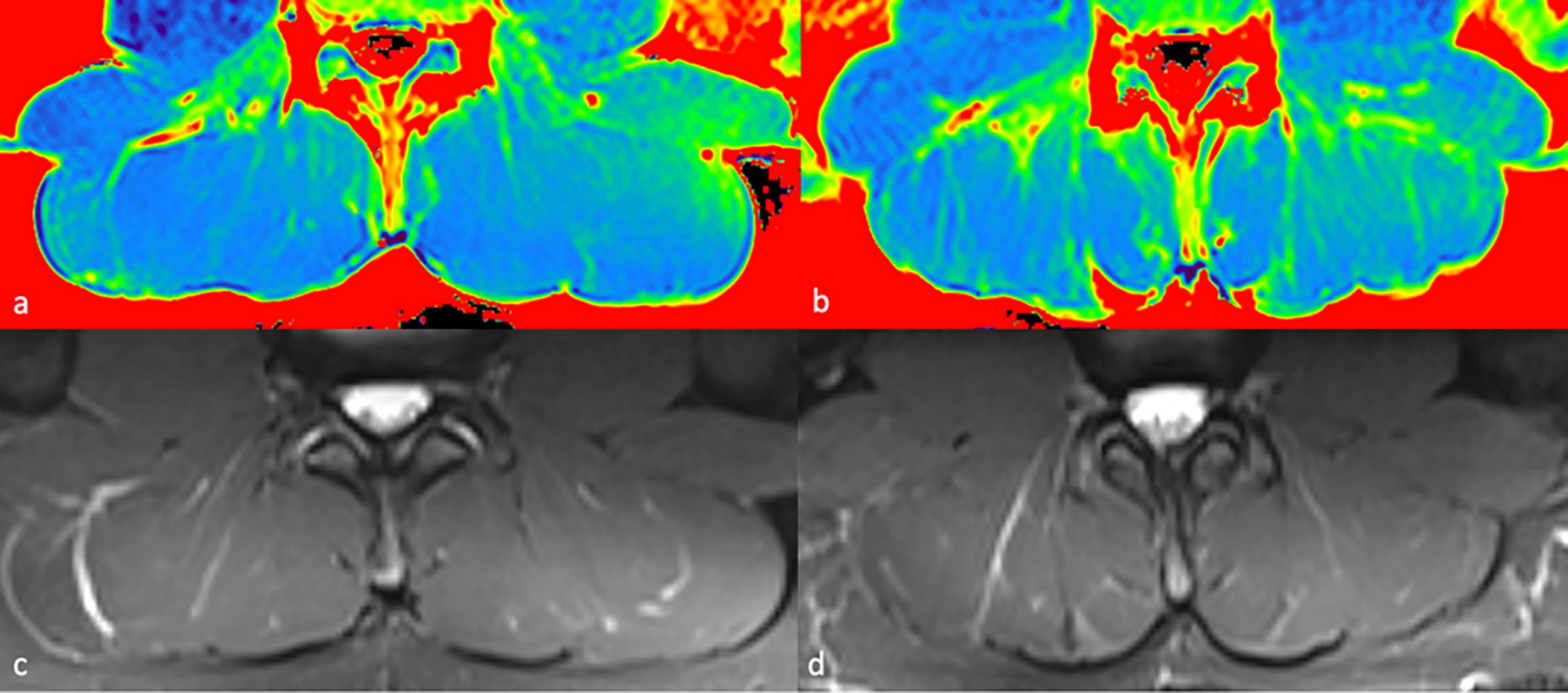
Color-coded ADC maps with corresponding fat-suppressed T2-weighted images. Exemplary color-coded ADC maps and T2-weighted inversion-recovery images of the lumbar spine of a 14-year old male scull rower (a,c) and a 15-year old male sweep rower (b,d). ADC values were significantly higher in sweep rowers. No pathological findings were evident on conventional images. ADC Apparent Diffusion Coefficient.

**Fig 3** shows exemplary ADC maps and corresponding T2-weighted inversion recovery images of a sweep and a scull rower.

**Fig 4:**
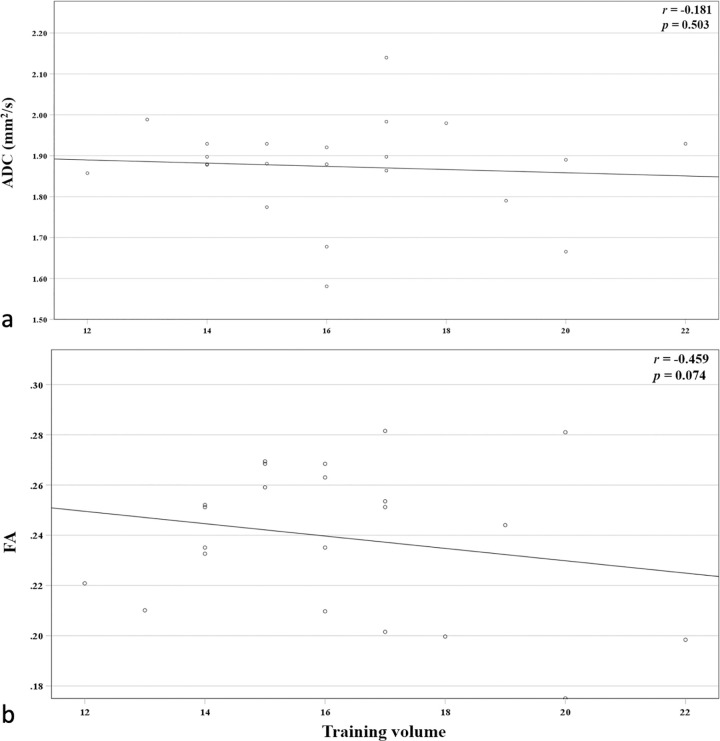
Correlation of DTI parameters with training volume. Scatter plots showing the correlation results of ADC values (a) and FA values (b) with training volume. There was no significant correlation for either DTI parameter. ADC, apparent diffusion coefficient; DTI, Diffusion Tensor Imaging; FA, fractional anisotropy.

No significant correlation was found between mean DTI parameters of the two autochthonous back muscles and training volume (r ≤ -0.459, p ≥ 0.074), see **Fig 4**.

## Discussion

This study used diffusion tensor imaging (DTI) in the lumbar spine muscles of adolescent elite rowers and examined changes in DTI parameters between different stroke typologies and correlated these parameters with the volume of training of the athletes.

The main finding of this study was that sweep rowers had higher total muscle diffusivity (apparent diffusion coefficient, ADC) and lower fractional anisotropy (FA) of the erector spinae and multifidus muscles compared to scull rowers.

Our study aligns with the post-exercise elevation of ADC and decrease of FA in the leg muscles of endurance athletes reported by Keller et al. and Froeling et al. [20; 26]. Similarly, an ADC elevation after isometric contraction has already been demonstrated in the erector spinae muscle [27]. Alterations of these DTI parameters correspond to less restricted diffusion of water and indicate cell swelling, interstitial edema, or disruption of membranes following microtraumatic muscular injury after strenuous exercise [19; 28]. Moreover, these correlate with histopathological signs of inflammation and repair [18].

The forces produced by eccentric muscle contraction are greater as those produced by isometric muscle contraction, suggesting that these are more likely to damage the muscle microstructure [25; 29]. Correspondingly, an ADC elevation and FA decrease after eccentric exercise has also been observed in the current literature [22–24]. Therefore, we hypothesize that the higher microstructural muscle damage observed in sweep rowers rather than in scull rowers is a result of excessive eccentric contraction, which can occurr during the catch phase of the rowing stroke [7]. Biomechanical differences between both stroke typologies have already been demonstrated with computerized motion analysis, showing that sweep ergometer rowing was associated with greater lumbar flexion compared to scull rowing and that flexion increased with the duration of rowing, most likely due to muscular fatigue [6]. Furthermore, our hypothesis would be consistent with a study by Strahan et al., which found that sweep rowers had a greater lateral bend as well as a greater magnitude of axial rotation at the catch compared to scull rowers [7]. In the present study, there was no significant association between training volume and DTI parameters. We assume this is mainly because the standard deviation of the training load in our study collective was relatively small and all athletes underwent the same training scheme in preparation of the rowing season. In contrast, a few studies have shown the dependency of DTI parameters to exercise intensity [30]. However, the impact of different training regimes on long-term DTI parameter changes has not yet been adequately investigated and could be of interest for future studies.

### Limitations

The first limitation of the present study is that the sample size was small and consisted only of male rowers. Therefore, the results of the present study should be interpreted with caution and require further validation. Second, although coaches and participants ensured that all rowers underwent the same training regimen, we cannot ensure that training intensity and/or duration was exactly the same for all participants, unlike in a laboratory setting. Third, we did not quantify exercise intensity with other outcome measures, which might affect the internal validity of our results. Other limitations include the lack of cross-over between stroke typologies (measuring DTI parameters of both athlete groups with both typologies) and the uncertainty of the clinical relevance of our findings. However, we believe that DTI has some potential in monitoring regeneration after muscular overuse injuries in athletes, although feasibility needs to be further improved.

## Conclusions

Our results indicate that the stroke typology might influence DTI parameters of the lumbar spine muscles. The observed alteration of DTI parameters are suggestive of microscopic tissue disruption and could be attributable to differences in underlying biomechanics of sweep and scull rowing.

## Supporting information

S1 Checklist STROBE statement—checklist of items that should be included in reports of observational studies. (DOCX)

S1 Data Minimal underlying dataset of this study. (XLSX)

## Abbreviations

ADC: Apparent Diffusion Coefficient
DTI: Diffusion Tensor Imaging
FA: Fractional Anisotropy
MRI: Magnetic Resonance Imaging
